# Sexual Activity in Adolescents and Young Adults through COVID-19 Pandemic

**DOI:** 10.3390/children8070577

**Published:** 2021-07-05

**Authors:** Androniki Stavridou, Chrysa Samiakou, Anastasia Kourti, Stauroula Tsiorou, Eleni Panagouli, Athanasios Thirios, Theodora Psaltopoulou, Theodoros N. Sergentanis, Artemis Tsitsika

**Affiliations:** 1 2nd Department of Pediatrics, “P. & A. Kyriakou” Children’s Hospital, School of Medicine, National and Kapodistrian University of Athens, 115 27 Athens, Greece; stavroniki@hotmail.com (A.S.); samichry@yahoo.com (C.S.); anastasiakourti.ak@gmail.com (A.K.); st.tsiorou@gmail.com (S.T.); eleni72000@yahoo.gr (E.P.); dunapower@gmail.com (A.T.); tsergentanis@yahoo.gr (T.N.S.); 2 Department of Clinical Therapeutics, “Alexandra” Hospital, School of Medicine, National and Kapodistrian University of Athens, 115 28 Athens, Greece; tpsaltop@med.uoa.gr

**Keywords:** COVID-19, sexual health, adolescents, young adults

## Abstract

During the COVID-19 pandemic, it has been postulated that the sexual life of adolescent and young adults has been impacted in various aspects, potentially affecting their well-being. Our aim is to investigate the potential changes in the sexual activity and relationships of adolescents and young adults during the COVID-19 pandemic. In general, a decrease in sexual desire was reported during the COVID-19 pandemic, in both genders. Fewer sexual intercourses and bonding behaviors between partners were associated with loneliness and depressive symptoms. On the contrary, an increase in sexual desire was expressed in a few people, with masturbation to be the most preferable means of satisfaction. The present paper highlights the multifaceted impact of COVID-19 upon the sexual life of adolescents and young adults during the ongoing pandemic. The changes observed in their sexual activity and relationships, could provide the basis of future preventive and educational programs.

## 1. Introduction

In December 2019, the first cases of COVID-19 were reported in Wuhan, China, raising concerns due to rapid spreading. The severe acute respiratory syndrome coronavirus 2 (SARS-CoV-2) is the underlying cause of the coronavirus disease (COVID-19), which has spread worldwide, forcing the World Health Organization (WHO) to declare it as a pandemic on 11 March 2020 [1,2]. Many countries imposed restrictive measures, such as lockdowns, social distancing, wearing masks, and frequent washing of hands through the day [3].

The fear of contacting the virus, as well as the public health measures, led people to diminish their visits to crowded places and thus many have faced difficulties in personal relationships, especially adolescents and young adults [4,5,6]. The lack of peer-to-peer contacts, due to home confinement and school closures, changed many aspects of life for youths. As a result, sexual activity, intimate relationships, access to contraception, protection from HIV, or other sexually transmitted infections (STIs) and physical, mental, or emotional well-being were adversely affected [7,8]. The social distancing that was imposed consequently led to the restriction of contacts between individuals, and therefore has affected the frequency of sexual activity, number of sexual partners, sexual desire and satisfaction, and the use of pornographic content [9]. According to the available literature, all these consequences seem to concern mainly younger individuals, such as adolescents and young adults [4,5,6,7,8,9].

The sexual life and activity of the above-mentioned age groups plays an important role in forming healthy sexual relationships and expressing their sexuality [8,9]. Sexuality constitutes a meaningful aspect of human development. The impact of COVID-19 in the sexual life of adolescents and young adults may significantly affect their sexuality. Thorough study of those effects will highlight the needs of youths during the pandemic and will provide substantial information in order to maintain their well-being. Thus, the purpose of this paper was to investigate the potential impacts and changes in the sexual activity and relationships of adolescents and young adults caused by the measures imposed due to the ongoing COVID-19 pandemic and the potential consequences to their sexuality in general.

## 2. Methods

A research of the literature was conducted in the following databases: Google Scholar, PsycInfo, PubMed, SCOPUS, and ERIC up to 14 June 2021. The algorithm used included: (sexual OR sex OR sexuality OR intimate) AND (health OR wellness OR life OR wellbeing OR well-being) AND (teens OR teenagers OR youngster OR youngsters OR adolescence OR adolescent OR adolescents OR “young adults” OR “younger adults” OR “Generation Z” OR juvenile OR juveniles OR youthful) AND (COVID-19 OR SARS-CoV-19 OR SARS-CoV-2 OR “2019-nCoV” OR “novel coronavirus”). The references of the eligible studies were searched through a snowballing technique, along with the relevant reviews.

The inclusion criteria were the following: Studies that examined changes in the sexual activity and relationships of adolescents and young adults (11–24 years old) during the ongoing COVID-19 pandemic were considered eligible. Studies that incorporated adolescents and young adults in a significant percentage were also included. Sexual activity must refer to sexual desire, frequency of intercourses, number of sexual partners, sexual satisfaction or dissatisfaction, and sexual preferences among adolescents and young adults. Concerning the type of study, case reports, cohort studies, cross-sectional studies, case series, and case-control studies, were included, while no language restrictions were imposed. The selection of studies was conducted by two authors (C.S. and A.S.) who worked independently. The following variables were used to extract data for each study: Title of the article, name of first author and year of publication, region/country where the survey was conducted, language, study period, study design, sample, sample size, age range and selection of sample, ascertainment and/or association with COVID-19 pandemic, outcomes, statistical analysis, and main findings. Quality assessment was performed in order to present accurate results. For this purpose the Newcastle–Ottawa Scale for cross-sectional studies [10] and cohort studies [11] were used.

## 3. Results

### 3.1. Selection of Studies

The research in the database retrieved 20,246 publications, while 1050 of them were duplicates; 16 studies (49,078 individuals) were finally considered eligible following the inclusion criteria imposed as mentioned above [12,13,14,15,16,17,18,19,20,21,22,23,24,25,26,27]. The eligible studies included data from various countries, three of them from China [12,13,14], four from the USA [15,16,17,25], while two presented data from UK [18,19], three from Italy [20,21,26], the remaining three from various countries, namely, France [22], Poland [23], and Turkey [24], while one included data from various countries [27]. The majority of them were cross-sectional studies (*n* = 10) and the remaining five were cohort studies, and only one study included mixed methods. Eleven studies referred to the COVID-19 pandemic or outbreak in general, whereas five studies referred to lockdowns (Table 1).

### 3.2. Sexual Activity and Behavior

According to the studies, a general decrease in sexual desire during the COVID-19 pandemic was reported from individuals [12,13,15,16,19,20,21,22,23,24]. More specifically, in a Chinese study conducted on youth (age range: 15–35 years old), 41% reported fewer intercourses, while 20% also reported a decrease in alcohol consumption before or during sex [12]. Men expressed more often a decrease in sexual partners (53%) compared to women (30%), while sexual satisfaction was reduced in both genders (32% of men and 39% of women), according to another Chinese study, in which the majority of the sample was 15–30 years old [13]. Gender and age differences could predict sexual dissatisfaction, along with COVID-19 potential infection during contact and occurrence of depressive symptoms, as stated by an Italian study by Cocci A et al. (mean age 21 years old) [20]. Following the above, another study in Italy (age range 18–40 years old (33.4% were 18–30 years)) presented a decrease in sexual intercourse by individuals during quarantine, mainly due to a lack of privacy and psychological stimuli [26].

According to Luetke et al., in the US, during home isolation and social distancing, conflicts were inevitable, leading to lower levels of sexual satisfaction mostly in men (a significant percentage (36.7%) in ages 18–39) [16]. Loneliness and depressive symptoms were associated with fewer sexual intercourses and bonding behaviors between partners in the US study by Hensel et al. (38.3% participants of ages 18–39) [15]. According to the Chinese study by Jianjun et al., women reported higher scores in the Sexual Compulsivity Scale (SCS) than men, while older counterparts also presented higher scores in SCS from younger ones (age range between 17 and 24 years), and individuals who live in urban areas reported lower scores in SCS than those living in rural areas [14]. The study by Fuchs et al. in Polish women (mean age: 25.1 years) reported a decrease in Female Sexual Function Index (FSFI) scores that was associated with the lack of work before and during the COVID-19 pandemic [23].

On the other hand, a small increase in sexual desire was expressed from men and women in China, Turkey, and France [13,22,24]. Masturbation was a preferable mean of satisfaction, through pornographic content during quarantine [12,20,22,25]. People who self-isolated from 0–5 days increased their sexual activity (33.5%) more than people who self-isolated for 11 days or more (47%) (32.4% were 18–34 years) according to a UK study. [19]. Higher knowledge of COVID-19 consequences was associated with more stable sexual behaviors among partners (a significant percentage (38.3%) in ages 18–39) [15]. According to the above, a significant number of individuals in an Italian study did not report a reduction in their sexual desire (71.3%, a large proportion of the sample being 18–30 years) [26].

Concerning couples, a decrease in sexual desire was reported in 10.4% of females and 9% of males in a French study (the majority of the sample were 15–30 years old) [22] while a Chinese study reported fewer instances of sexual intercourse by both genders (which also had a majority of the sample in the 15–30-year age group) [13]. Having children under five led to greater instances of sexual release, while having older children (elementary aged) lead to a decrease in those behaviors, as evidenced by Hensel et al. in the USA [15]. Although, many couples reported no difference in their sexual activities in general, in an Italian cross-sectional study (61.5% were under 34 years old), 12.1% of men and 18.7% of women stated that there was an increase in their sexual desire [21].

The use of technology, including dating or hooking-up apps, decreased, as evidenced in a study on 15–40+-year-old US men having sex with men [17]. Those apps were used by young people, in order to stay in touch, but not in face-to-face interactions. Thus, the opportunities for sexual intercourse were limited. The reduction of sexual activity among young people (under 17 and over 18 years old), could be reflected in the reduction in demand for sexual health services (SHS) as stated by Thomson-Glover et al. in the UK [18]. Findings support the above data were also presented in a study including Adolescents Sexual Minority Men (ASMM) (14–17 years old), who used social media and virtual means of communications to stay in touch with their sexual partners [25]. In addition, in the same context, in a study including Gay, Bisexual, and Other Men Who Have Sex With Men (GBMSM) (age range 18–35 years old), the use of social media for communication was reported, while an impact on sexual life during the ongoing COVID-19 pandemic was expressed [27].

### 3.3. Risk of Bias

The majority of studies were cross-sectional (*n* = 10) and six of them scored high (either 9 or 8) in the Newcastle–Ottawa scale. In some cases, the selection of the sample was detailed, but in six of them, the non-responder rate was not justified. The ascertainment of the exposure was implemented through online questionnaires, due to COVID-19 restrictions, and thus were not always validated. Although the control of confounders was performed through appropriate statistical analysis, the outcome was mainly assessed through self-report questionnaires and could not be totally reliable. Furthermore, the five cohort studies provided good (*n* = 2) and fair quality (*n* = 3).

## 4. Discussion

The ongoing COVID-19 pandemic seems to have multiple effects in the sexual life of youth. Adolescents and young adults are the age groups who might be less vulnerable to the virus, but seem to suffer greatly from psychosocial consequences [28]. Social distancing, school closure, and restriction of activities lead to a reduction of any kind of social contact. Additionally, as they were obliged to stay most of the time inside, adolescents and young adults were subjected to increased parental monitoring, which reduced independence, physical interaction with peers, and privacy [29]. Subsequently, according to available reports, a decrease was observed in age groups concerning partnered sex, sexual behaviors, and relationships [4,29]. On the other hand, an increase was recorded in online social connections, as 65% of teenagers used texting or interaction via social media more often than usual [29].

According to our findings, a decrease in sexual desire during the COVID-19 pandemic, concerning fewer intercourses, was reported in general, due to loneliness and depressive symptoms, which were also associated with fewer sexual intercourses [12,13,15,16,19,20,21,22,23,24,25,26,27]. Sexual partners decreased, especially concerning males, while sexual satisfaction was reduced in both genders (32% of men and 39% of women) in the age group of 15–30 years old [13].

Additionally, according to our findings, the knowledge of COVID-19 consequences was associated with more stable sexual behaviors among partners in ages 18–39 [15]. Our study also detected that the use of dating or hooking-up apps decreased during COVID-19 restrictions, according to a study on men having sex with men (age range 15–40 years old) [17]. Nevertheless, online connections seem to be rather important, as they offer options to connect despite the social distancing and stay-at-home orders [29]. Another finding was the reduction in demand for sexual health services, mainly due to fear of infection, among young people [18], possibly associated with problems in accessing condoms, HIV and STI testing, and treatment services, leading to increased rates of sexually transmitted infections and unintended pregnancy among youth [4]. For adolescents and young adults considered to be vulnerable, especially those who are part of the LGBTQ community, who had to face discrimination, violence, and lack of access to health, the COVID-19 pandemic created a more hostile environment for them. Thus, deterioration in their mental health and well-being was noted, including their sex life [30,31].

On the other hand, during COVID-19 and due to the circumstances, methods such as telehealth, home-based sexually transmitted infections screening, and contraceptive delivery were developed [4]. According to the literature, these methods were considered safe and effective and acceptable to youth. Thus, some of them could be adopted afterwards in order to provide better health care concerning the sexual health of these age groups [4]. Inaccessibility of sexual and reproductive healthcare services seems to be one of the main causes of increased rates of sexually transmitted infections among adolescents and young adults, a fact that was also highlighted during the pandemic [4,18].

Furthermore, it seems that the COVID-19 pandemic has exposed adolescents and mostly girls to multiplied risks concerning their sexual health, such as sexually transmitted infections including HIV and Human Papilloma Virus, as well as unintended pregnancies [30]. According to reports, an increase was also observed in sexual and gender-based violence, a fact that is probably the result of the difficulty in accessing relevant services such as intervention programs [30]. Thus, the pandemic seems to have additionally highlighted the need for better organization and the development of youth-friendly and easy-to-access sexual and reproductive healthcare services. Concerning the limitations of this study, the COVID-19 pandemic is an ongoing phenomenon, and thus, the impact in the sexual activities of adolescents and young adults need to be further tested. In order to provide a global understanding of relevant effects, an examination of diverse groups should be a priority. Confinement measures and social distancing created a more complex reality, affecting all aspects of social life; the interplay with sexual life should be monitored in a more systematic way. Furthermore, the majority of the studies were cross-sectional, providing no long-term results, while self-reported assessments limited the validity of studies.

## 5. Conclusions

In conclusion, the present paper highlights that sexual activity, an important aspect of adolescents’ and young adults’ life, was reported to be considerably affected during the ongoing COVID-19 pandemic. The changes observed in sexual activity and relationships could play an important role in forming preventive and educational programs in collaboration with parents, caregivers, teachers, and medical staff, aimed towards good sexual health and well-being.

## Figures and Tables

**Table 1:** Description of studies examining sexual health in adolescents and young adults during the COVID-19 pandemic.

First Author (Year)	Country	Language	Study Period	Study Design	Sample	Sample Size	Age Range	Selection of Sample	Ascertainment and/or Association with the COVID-19 Pandemic	Outcomes, Way/Questionnaires They Were Measured	Statistical Analysis	Main Findings
Li et al. (2020) [12]	China	English	1–5 May 2020	Cross–sectional	Users of an online questionnaire survey platform	967 (541 males)	15–35	15–35 years old, living in China and reported penetrative sex	COVID-19 pandemic	Online survey about sexual and reproductive health	Logistic regression analysis, Chi–square test	A total of 212 (22%) reported a decrease in sexual desire, 396 reported (41%) a decrease in the frequency of sex, 192 reported (20%) a recent decrease in alcohol consumption before or during sexual activities, and 94 (10%) reported a decrease in risky sexual behavior. A total of 298 (31%) reported partner relationship deterioration during the pandemic, 291 (30%) reported an increase in masturbation during the pandemic, and 227 (23%) reported an increase in the use of pornography. Accommodations during the pandemic, exclusive relationship status, sexual desire, and sexual satisfaction were closely related to partner relationships.
Li W. et al. (2020) [13]	China	English	13–15 March 2020	Cross–sectional	Users of Han Chinese ethnicity of social media platforms	459 (270 males)	18–45	Participants aged 18–45 years, with a history of sexual activity	COVID-19 pandemic	12–item online questionnaire assessing present and previous sexual behaviors	Descriptive statistics and logistic regression analysis	A reduction in sexual desire was experienced (25%) while 44% reported a decrease in the number of sexual partners, with men reporting more (53% vs. 30%). Lower frequency in sexual intercourse was reported by 37%, including 26% married men and 28% married women. From married men (49%) and married women (29%) a decrease in the number of sexual partners was reported. A total of 32% of men and 39% of women experienced a reduction in sexual satisfaction and in risky sexual behaviors. Only 18% of men and 8% of women experienced increased sexual desire.
Landry S. et al. (2020) [22]	France	French	7th and the 8th week of the first lockdown in France	Cohort	Users of LinkedIn, Facebook, and Twitter	844 (290 males) (couples *n* = of 437, 143 are males), (singles = out of 407, 147 are males)	15–70 years old. 71.2% of self-confined couples were 15–30-year-olds and 88.2% of singles were 15–30-years-old.	Participants aged 15–70 years, with a history of sexual activity	Lockdown	Two questionnaires, one for participants’ confining themselves in couple, one for participants’ confining themselves as singles, assessing the demographics and comparing present (during lockdown) with previous sexual behaviors	Descriptive statistics and frequency measurements	Changes in sexual habits were reported in both couples and those without partners during this period. A decrease in sexual desire was reported in 10.4% of females and 9% of males.
Hensel D. et al. (2020) [15]	USA	English	10–20 April 2020	Cross–sectional	Users of a web research panel	1010 (48.5% males)	18–94 (a significant percentage 38.3% in ages 18–39)	Representative of the overall US population	COVID-19 pandemic	The 26-item online survey assessing demographics, especially the predictor children in home and self–reported past month changes in 10 solo and partnered sexual behavior categories. Five items for the past month of depressive symptoms and three items for loneliness.	Multinomial logistic regression	In the past month of sexual behavior, half of all adults reported change, mostly a decrease. The younger the age of their children (under the age of five), the greater the likelihood of stability and/or increased partnered behaviors, whereas having elementary-aged children was often associated with a decrease in these behaviors. Past month of depressive symptoms and loneliness were associated with both reduced partnered bonding behaviors and partnered sexual behaviors. The greater the perceived risk of COVID-19, the lesser the reported solo and partnered sexual behaviors. Stability in partnered sexual behaviors was reported in people with greater COVID-19 knowledge.
Luetke, M. et al. (2020) [16]	USA	English	10–20 April 2020	Cross–sectional	Users of a web research panel	742 (49% males)	18–94 (a significant percentage 36.7% in ages 18–39)	Representative of the overall U.S. population	COVID-19 pandemic	The 26-item online survey assessing demographics and self-reported past month changes in 10 solo and partnered sexual behavior categories. Five items for the past month of depressive symptoms and three items for loneliness. Two other secondary outcomes were assessed: Orgasm and emotional closeness at last sexual event	Chi-square tests, student’s t-tests, and multinomial logistic regression	A decreased frequency in several intimate and sexual behaviors was associated with conflict, which also tended to be associated with lower levels of self-reported orgasms and emotional connectedness at participants’ most recent past month sexual events. The association between conflict and decreased frequency of the intimate and sexual behaviors tended to be stronger among men than among women. Greater difficulties were reported in romantic and/or sexual relationships.
Sanchez T. et al. (2020) [17]	USA	English	2–13 April 2020	Cross–sectional	Participants of the American Men’s Internet Survey (AMIS) who conducted mailed HIV/STI testing the past 2 years	1051	15–40+	15 years or older, male sex at birth, resided in the US, and reported oral or anal sex with a man at least once at any time in the past	COVID-19 pandemic	Online questionnaire	Differences in the prevalence of adverse COVID-19-related impacts by participant age were reported as bivariate prevalence ratios (PR) and 95% confidence intervals (CI)	The majority of the participants reported fewer sex partners and opportunities to have sex due to COVID-19 and a decrease or unchanged usage of dating or hook-up apps, mainly to connect with other men. Condom access and usage were unchanged due to COVID-19. One-tenth of participants had increased use of recreational drugs and one-quarter had increased alcohol consumption.
Thomson–Glover R. et al. (2020) [18]	UK	English	10 February to 22 March 2020 (pre–lockdown) AND 23 March to 30 April 2020 (during lockdown)	Cohort	Young people	25,651 (24,910 attendances for sexual health services and 741 emergency contraception attendances)	17 and under and 18 and over	Sexual health attendances among young people (YP) within a semirural service setting and at services based in London and Surrey	Lockdown	Hospital records	NR	Due to COVID-19 restrictions, a reduction in demand for sexual health services (SHS) may reflect reduced sexual activity and partnerships among young people.
Jianjun et al. (2020) [14]	China	English	NR	Cross–sectional	Undergraduate students	3219 (714 males)	17–24	Recruited online via smartphone from universities	COVID-19 outbreak	Adolescent Self–Rating Life–Events Check List (ASLEC), and 8 psychological behaviors indicators assessed individual subjective emotion for the COVID-19 stress—Items, Perceived Social Support Scale (PSSS)—Sexual Compulsivity Scale (SCS)	SPSS 22.0 and MPLUS 7.0—differences in testing, descriptive statistics, correlation analyses, hierarchical regressing analyses, and the testing of mediator (moderator) model	Females reported higher COVID-19-related stress, PSSS, and SCS than males. In particular, COVID-19 stress (*p* < 0.001) and PSSS (*p* < 0.01) was significant. Participants over 21 had lower COVID-19-related stress, and higher PSSS and SCS compared to younger people, with the differences of COVID-19 stressing (*p* < 0.001) being significant. Urban participants had higher COVID-19 stressing, and lower PSSS and SCS compared to rural participants, while only COVID-19-related stress (*p* < 0.001) and SCS (*p* < 0.05) differences were significant. Therefore, these results showed that differences in COVID-19 stress, PSSS, and SCS were significant for gender, years, and urban/rural status.
Cocci A. et al. (2020) [20]	Italy	English	March to April 2020	Cross–sectional	From social networks (Instagram and Facebook)	1515	Mean age = 21	Posts on social networks (Instagram and Facebook), through Google Forms	Lockdown	The Beck Depression Inventory (BDI–II) and the Beck Anxiety Inventory (BAI) in the Italian validated version, sexual satisfaction was evaluated by asking the question “Overall, how satisfied are you with your sex life during quarantine on a scale ranging from 1 (not at all satisfied) to 5 (extremely satisfied)?”	Multivariate logistic regression analysis, BDI score, BAI score, Chi-square test	Median BDI score was 10.0 (IQR: 5.0–16.0) and median BAI score was 11.0 (IQR: 6.0–20.0). A total of 602 (39.74%) respondents answered that quarantine increased autoerotism more than before. Although only 7.46% of the participants reported “no satisfaction” before the quarantine, 53.53% complained about that during quarantine (*p* < 0.01). Women had greater depression (BDI–male: 8.0 [IQR 4.0–13.0]; BDI–female: 11.0 [IQR 6.0–17.0]; *p* < 0.01) and anxiety versus males (BAI–male: 7.0 [IQR 3.0–14.0]; BAI–female 13.0 [IQR 7.0–23.0]; *p* < 0.01). Multivariate logistic regression analysis, presented that age (odds ratio [OR]: 0.96; *p* < 0.01) and BDI (OR: 1.07; *p* < 0.01) in males and age (OR: 0.96; *p* < 0.01), BDI (OR: 1.03; *p* < 0.01) and “knowing people positive at COVID” (OR: 0.78; 0.05) in women were significant predictors of sexual dissatisfaction.
Panzeri M. et al. (2020) [21]	Italy	English	11 April to 5 May 2020	Cross–sectional	Cohabiting couples	124 (26.6% males)	23–60 (61.5% under 34)	Online survey	Lockdown	Online questionnaire including Quality of Marriage Index (QMI)—Two factors of the Sexual Desire Inventory (SDI)—Three factors of the Depression Anxiety Stress Scales—21 (DASS–21)—The total score of the Patient Health Questionnaire (PHQ–15)	Logistic regression analysis, t–test analysis, and Chi–square analysis	Most couples responded that they did not perceive any differences in their sexuality, despite the pandemic’s consequences. Some female participants reported a decrease in pleasure, satisfaction, desire, and arousal. The main reasons for this seemed to be worry, lack of privacy, and stress. Even though participants seemed to show high levels of resilience, the negative aspects of lockdown could affect their quality of sexual life.
Yuksel B. & Osgor F. (2020) [24]	Turkey	English	1 February 2018 to 30 September 2019 and 11 March to 12 April 2020	Cohort	Women patients in Haseki Training and Research Hospital and Esenler Maternity and Children’s Hospital	58	Mean age = 27.6	Patient data from the electronic data system	COVID-19 pandemic	Female Sexual Function Index (FSFI) questionnaire	The Kolmogorov–Smirnov test, the paired sample t-test and McNemar test were used to compare the averages and percentages	During the pandemic, the average frequency of sexual intercourse was significantly increased compared with 6–12 months prior (2.4 vs. 1.9, *p* = 0.001). Female Sexual Function Index (FSFI) scores were significantly better before the pandemic compared with scores during the pandemic (20.52 vs. 17.56, *p* = 0.001).
Fuchs A. et al. (2020) [23]	Poland	English	March to April 2020	Cohort	Research in Hospital and Esenler Maternity and Children’s Hospital	764	Mean age = 25.1	Patients of Department of Pregnancy Pathology, Department of Woman’s Health, School of Health Sciences in Katowice, Medical University of Silesia	COVID-19 pandemic	Female Sexual Function Index (FSFI) questionnaire	Wilcoxon’s rank test, comparison Chiˆ2, Kruskal–Wallis test, U Mann–Whitney test	The overall FSFI score before the pandemic was 30.1 ± 4.4 and during, it changed to 25.8 ± 9.7. Desire, arousal, lubrication, orgasm, satisfaction, and pain scores decreased as well (*p* < 0.001). There was statistically significant association between the workplace and the change of FSFI scores before and during the COVID-19 pandemic (*p* < 0.01). The largest decrease in FSFI score was noticed in the group of women who did not work at all (5.2 ± 9.9).
Jacob L. et al. (2020) [19]	UK	English	17 March 2020	Cross–sectional	Adults aged 18 years and over, currently residing in the UK and self-isolating/social–distancing due to COVID-19	868 (male 36.9%)	18–75 (32.4% 18–34 years)	Participants were recruited through social media and through national media outlets (BBC, 26 March 2020) and by distributing an invitation to participate through existing researcher networks	COVID-19 pandemic	Online survey	Multivariable logistic regression, chi-squared tests for categorical variables and t-tests for continuous variables—analysis of variance (ANOVA), Cramer’s V, Cohen’s d, regression analysis, and logistic regression analysis	A total of 39.9% of the sample reported engaging in sexual activity at least once per week on average, was classified as sexually active during lockdown. The mean number of sexual activities was 1.75 in the overall population, and was significantly higher in men than women. The prevalence of sexual activity significantly increased from 33.5% in people who were self-isolated for 0–5 days to 47.0% in those who were self-isolated for 11 days. Adults with sexual activity were mostly male and of a younger age; married/in a domestic relationship, employed, having high annual household income, and consuming alcohol, while the number of chronic physical conditions was significantly lower in the sexually active than in the non-sexually active group.
Nelson K.M. et al. (2020)	US	English	27 March 2020 to 8 May 2020	Mixed method	Adolescents Sexual Minority Males (ASMM)	151	14–17	Online survey	COVID-19 pandemic	Demographics, questions concerning sexual preferences, and sexual behavior	Fisher’s exact and t-tests, framework matrix analysis	The participants reported that they saw their sexual partners less often, while watching pornography and masturbating was more often. Sexting and messaging through hook-up apps and websites were used more frequent during the last three months. Social distancing made physical connection more difficult and an increase in the use of virtual means of communication was reported, such as video chatting.
Cito G. et al. (2021)	Italy	English	April 2020	Cohort	Young participants	1576 (64.6% women)	18–40 (33.4% 18–30)	Online survey	Lockdown	Self-administered questions on couples’ sexuality and on individual sexual behaviors	Chi-square test or Fisher’sexact test, Mann–Whitney U test or Student’s t-test	Sexual intercourse decreased significantly during quarantine. The main reasons were: Poor privacy (43.2%) and lack of psychological stimuli (40.9%). On the other hand, a significant number of participants (71.3%) did not report a reduction in sexual desire. A total of 61.2% did not report, also, a reduction in autoerotism. Accordingly, those reporting a decrease in masturbation was mainly due to poor privacy (46.4%) and lack of desire (34.7%). Men presented lower sexual desire during quarantine, than women (*p* < 0.01).
Holloway I.W. et al. (2021)	Multiple countries	English	16 April 2020 to 24 May 2020	Cross–sectional	Gay, Bisexual and Other Men Who Have Sex With Men (GBMSM)	10079	18–35	Online survey via Hornet	COVID-19 pandemic	58-question online survey regarding the impact of COVID-19 on a variety of health and mental health outcomes	Chi-squared tests, multivariable logit regressions	GBMSM who practiced physical distancing were more anxious (aOR = 1.37), lonely (aOR = 1.36), and reported that their sex life was impacted during the pandemic (aOR = 2.95), while a small number reported satisfaction with their sex life at the time (aOR = 0.76). Social media and technology was more likely to be used by those who practiced physical distancing.

## Data Availability

Data is contained within the article.
